# Complete Pathologic Response Following Neoadjuvant Chemoradiotherapy and High-Dose-Rate Brachytherapy for Locally Advanced Endometrial Carcinoma

**DOI:** 10.7759/cureus.407

**Published:** 2015-12-15

**Authors:** Zachary Horne, John A Vargo, John T Comerci, Sushil Beriwal

**Affiliations:** 1 Department of Radiation Oncology, University of Pittsburgh Cancer Institute, Pittsburgh, USA; 2 Department of Obstetrics and Gynecology, Division of Gynecologic Oncology, Magee Women's Hospital of University of Pittsburgh Medical Center, Pittsburgh, USA

**Keywords:** hdr brachytherapy, brachytherapy, endometrial cancer, neoadjuvant chemoradiation, complete response

## Abstract

The majority of uterine cancers present at an early stage because of abnormal post-menopausal bleeding. Locally advanced endometrial carcinoma with extension to the parametria represents a rare subset of patients with treatment options involving neoadjuvant therapy followed by extrafascial hysterectomy or definitive non-operative management. Neoadjuvant therapy enables R0 resection in the majority of patients but with relatively low rates of complete pathologic response. Herein, we detail the case of a patient with bulky FIGO Stage IIIB endometrial carcinoma with pelvic and para-aortic lymphadenopathy who achieved a complete response with neoadjuvant chemoradiotherapy and high-dose-rate brachytherapy prior to extrafascial hysterectomy.

## Introduction

Most uterine cancers present at an early stage because of abnormal uterine bleeding in post-menopausal women [1]. Locally advanced (FIGO Stage III-IVA) endometrial carcinoma with extension to the parametria is a relatively rare presentation of the disease, representing less than 10% of all uterine cancers [1]. Prior to the FIGO paradigm change to surgical staging in 1988, preoperative radiotherapy was the standard for all locally advanced disease, as even a radical hysterectomy would not result in an R0 resection [2-3]. Surgical staging has since become the standard of care for the majority of patients, but neoadjuvant therapy followed by an extrafascial hysterectomy remains the standard for patients with parametrial involvement [4].

We previously reported on our institutional outcomes of preoperative radiotherapy and brachytherapy with or without systemic chemotherapy in 36 patients with locally advanced endometrial carcinoma and clinical involvement of the cervix and/or parametria [5]. While all patients had clinical evidence of cervical involvement, 50% had parametrial involvement and 17% had para-aortic lymphadenopathy. Of the patients treated, 92% were able to proceed with surgical resection, 100% of whom achieved an R0 resection rate with a pathologic complete response of 24%, indicating that the regimen is effective for the conversion of otherwise inoperable patients with parametrial extension. Herein, we report the case of a patient with bulky, locally invasive FIGO Clinical Stage IIIB endometrial carcinoma with pelvic and para-aortic lymphadenopathy treated with preoperative chemoradiotherapy and brachytherapy who achieved a complete pathologic response, highlighting the potential clinical efficacy of such an approach in the uncommon endometrial cancer patient who presents with extensive disease. The University of Pittsburgh Medical Center approved this study and issued approval #PRO13020306. Informed consent was waived under our institutional IRB approval.

## Case presentation

**Figure 1:**
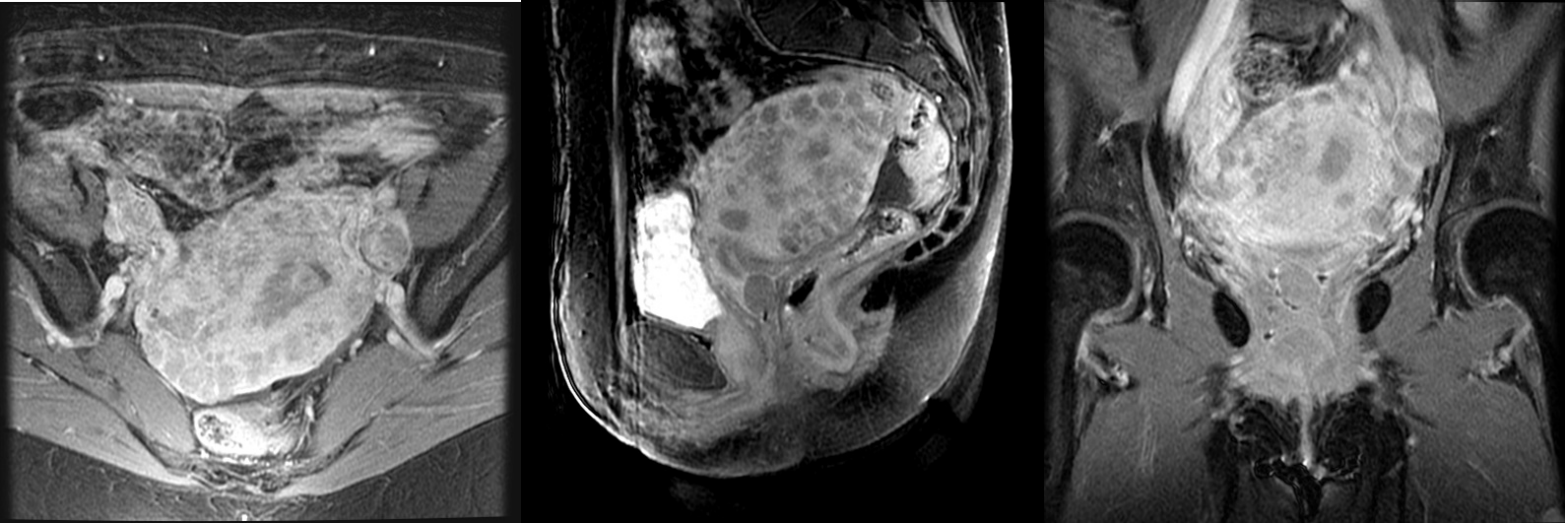
MR imaging at the time of presentation showing bulky, locally advanced disase. Axial, sagittal, and coronal images at the time of diagnosis showing an enlarged uterus measuring 9.6 x 8.6 x 7.7 cm with a polypoid endometrial mass measuring 2.0 x 1.6 cm and an endometrial stripe up to 1.8 cm. Extension to the lateral sidewall was noted bilaterally as well as extension to the lower uterine segment and upper cervix.

The patient was a 60-year-old female with no significant prior medical history who presented to the emergency department complaining of abdominal pain. The physical exam revealed a lobulated, friable soft tissue mass protruding into the cervical canal with extension through the lateral parametria to the pelvic side walls. She underwent a CT of the abdomen and pelvis, which showed an enlarged uterus with a thickened endometrial stripe. Transvaginal biopsy confirmed FIGO Grade III endometrial endometrioid carcinoma. A follow-up pelvic MRI confirmed a 9.6 x 8.6 x 7.7 cm uterine mass with extension to the cervix, bilateral lateral parametria, and pelvic side walls with suspicion of sigmoid colon involvement (Figure 1).

An 18-FGD PET/CT scan showed no evidence of distant metastases but revealed FDG-avid left external, right common, aortocaval, para-aortic, and retrocrural lymphadenopathy consistent with Clinical Stage IIIB disease.

**Figure 2:**
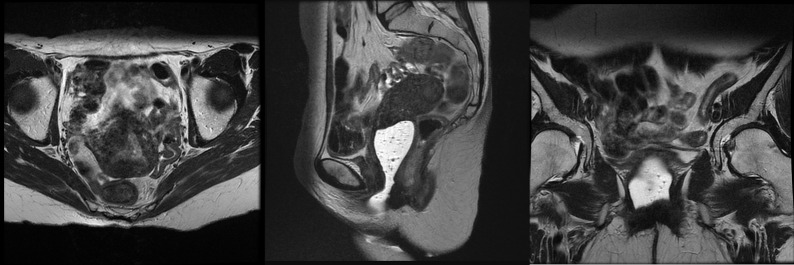
MR imaging following neoadjuvant chemoradiotherapy showing a near complete response. Axial, sagittal, and coronal images taken at the completion of concurrent chemoradiotherapy, prior to high-dose-rate brachytherapy. A 0.8 x 0.7 cm nodule was noted on the right side of the endometrial cavity, representing residual disease. All previously noted pelvic and retroperitoneal lymphadenopathy had resolved.

Because the disease invaded through the bilateral parametria to the pelvic sidewalls, surgical resection, even with a radical hysterectomy, was not an option. Therefore, in an attempt to make her a candidate for surgical resection, she underwent a course of neoadjuvant chemoradiotherapy with pelvic and para-aortic field IMRT and concurrent low-dose weekly cisplatin. Using a simultaneous integrated boost technique, she received 45 Gy to the pelvic and para-aortic chain and 55 Gy to the PET-avid lymphadenopathy, all in 25 fractions. A repeat pelvic MRI to delineate the extent of disease prior to brachytherapy showed an excellent response with a small nodule of residual disease measuring 0.8 x 0.7 cm in the right side of the endometrial cavity (Figure 2).

**Figure 3:**
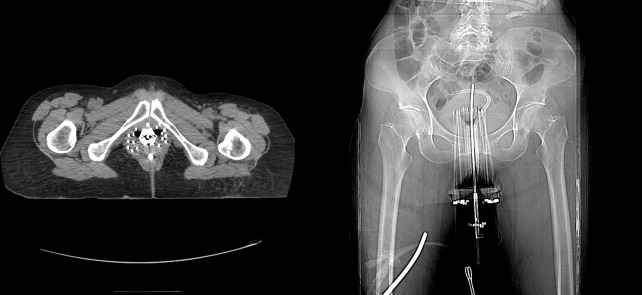
Planning CT for interstitial high-dose-rate brachytherapy following neoadjuvant chemoradiotherapy. Axial and scout images taken immediately following the placement of interstitial needles through the cervix with a central intra-cavitary tandem for high-dose-rate brachytherapy.

She subsequently was taken to the operating room for the placement of an intrauterine tandem and interstitial needles based on the initial extent of her disease (Figure 3), through which an additional 15 Gy in three fractions was delivered via a high-dose-rate iridium-192 source over a period of 36 hours as a preoperative course.

She tolerated her treatment well without any severe adverse GI or GU side-effects.

After restaging CT imaging that showed no evidence of disease, she was taken to the operating room one month following completion of her neoadjuvant therapy for an extrafascial hysterectomy, bilateral salpingo-oophorectomy, omentectomy, and pelvic washings. Pathology showed no residual carcinoma and only therapy-related changes. Because of her initial extent of local and nodal disease, she was maintained on monthly bevacizumab infusions for a total of 18 cycles. She remains clinically and radiographically without disease 18 months from her surgery.

## Discussion

Outcomes for FIGO Clinical Stage III endometrial carcinoma remain poor, with five-year survival rates ranging from 50-66% [1]. Patients with endometrial carcinomas which invade the parametria are not upfront surgical candidates because of the inability to attain negative margins, even with a radical hysterectomy. Previous institutional series have shown excellent survival rates with a preoperative regimen utilizing external beam radiotherapy with or without chemotherapy and low-dose-rate brachytherapy, followed by surgical management [2-3]. Therefore, neoadjuvant therapy to enable a resection is the standard of care with a Category 2B recommendation because of a lack of randomized evidence [4, 6]. The alternative approach to neoadjuvant therapy followed by resection is definitive non-operative management with external beam radiotherapy and brachytherapy [6-8]. Distant failure also remains an issue for patients treated with radiotherapy alone, arguing for the necessity of adjuvant chemotherapy [9].

When patients are medically fit for surgery, our institution utilizes the neoadjuvant approach with high-dose-rate brachytherapy and attains a rate of R0 surgical resection greater than 90% [5]. The patients from our series, 50% of whom had parametrial involvement and 17% of whom had para-aortic lymph node involvement, achieved excellent survival rates. Even with excellent local control, however, distant failures remain an issue for these patients, especially those with nodal disease at presentation, and therefore, chemotherapy is the backbone of their treatment regimen [4, 10].

## Conclusions

Neoadjuvant chemoradiotherapy followed by high-dose-rate brachytherapy and extrafascial hysterectomy represents a reasonable treatment algorithm in patients with locally advanced disease and para-aortic lymphadenopathy.
